# P08 Improving antibiotic stewardship in paediatric appendicitis: a quality improvement project

**DOI:** 10.1093/jacamr/dlag102.014

**Published:** 2026-06-26

**Authors:** Daniel Andreyev, Eduardo Villatoro

**Affiliations:** Sherwood Forest Hospitals Trust, Mansfield, UK; Sherwood Forest Hospitals Trust, Mansfield, UK

## Abstract

**Background:**

Appendicitis is one of the most common causes of paediatric surgical admission. Despite its frequency, adherence to antibiotic guidelines is often inconsistent. Inappropriate antibiotic use in children is associated with increased antimicrobial resistance, higher risk of secondary complications such as *Clostridioides difficile* infection and asthma, and prolonged hospital stays. BSAC provides clear recommendations for antimicrobial management in both uncomplicated and complicated paediatric appendicitis.

**Objectives:**

This study assessed local adherence to national guidance and evaluated the impact of a targeted educational intervention. We aimed to improve antibiotic stewardship and ultimately improve patient outcomes.

**Methods:**

A retrospective cohort study was conducted comparing two groups of paediatric patients undergoing appendicectomy over consecutive two-month periods. Cohort A represented pre-intervention practice, while Cohort B represented post-intervention outcomes. The intervention consisted of a structured departmental teaching session for medical and nursing staff, supported by visual reminder posters displayed within the paediatric surgical department. For both cohorts, we evaluated adherence to national antibiotic guidelines in the pre- and post-operative setting, duration of antibiotic therapy, timing of post-operative blood tests to guide IV-to-oral switch, length of stay, and infection-related readmissions.

**Results:**

In Cohort A, overall adherence to guidelines was 26%. While 45% of uncomplicated cases were managed appropriately, no patients with complicated appendicitis received recommended guideline-based therapy. Broad-spectrum antibiotic (piperacillin/tazobactam, Tazocin) was frequently used inappropriately. Only 25% of complicated cases had appropriately timed post-operative blood tests, and 87.5% received prolonged antibiotic courses. Infection-related readmission occurred in 12.5% of cases. Following intervention (Cohort B), overall adherence improved substantially. Appropriate management increased to 60% in uncomplicated cases and 40% in complicated cases. Tazocin use aligned fully with guideline indications. Seventy percent of complicated cases received the correct duration of antibiotic therapy, 50% had appropriately timed post-operative blood tests facilitating earlier IV-to-oral switch, and no infection-related readmissions were observed. Length of stay was reduced in patients whose management adhered to guideline-directed blood monitoring and antibiotic duration.

**Conclusions:**

This simple, low-cost educational intervention significantly improved adherence to national antimicrobial guidelines in paediatric appendicitis. This was associated with more appropriate antibiotic selection and duration, improved stewardship practices, reduced prolonged hospitalization, and elimination of infection-related readmissions within the study period. This is essential in a paediatric cohort with worried families and distressed children. Structured education and visible reminders represent effective strategies to enhance antimicrobial stewardship in common paediatric surgical conditions.

